# Complete genome sequence of *Streptococcus intermedius* strain XH2169

**DOI:** 10.1128/mra.00052-24

**Published:** 2024-08-20

**Authors:** Yisha Zhang, Yue Yao, Feng Zhao, Yunsong Yu, Xiaoting Hua

**Affiliations:** 1 Department of Infectious Diseases, Sir Run Run Shaw Hospital, Zhejiang University School of Medicine, Hangzhou, China; 2 Key Laboratory of Microbial Technology and Bioinformatics of Zhejiang Province, Hangzhou, China; 3 Regional Medical Center for National Institute of Respiratory Diseases, Sir Run Run Shaw Hospital, School of Medicine, Zhejiang University, Hangzhou, China; 4 Department of Clinical Laboratory, Sir Run Shaw Hospital, Zhejiang University School of Medicine, Hangzhou, Zhejiang, China; 5 Key Laboratory of Precision Medicine in Diagnosis and Monitoring Research of Zhejiang Province, Hangzhou, Zhejiang, China; University of Maryland School of Medicine, Baltimore, Maryland, USA

**Keywords:** *Streptococcus intermedius*, whole-genome sequencing

## Abstract

Here, we report the complete genome sequence of *Streptococcus intermedius* strain XH2169 isolated from a blood sample in China. The genome comprises a single circular chromosome with a length of 1,944,282 bp. It harbored 1,891 coding gene sequences, 16 rRNA genes, 60 tRNA genes, and 3 noncoding RNA genes.

## ANNOUNCEMENT

*Streptococcus intermedius* is a β-hemolytic Gram-positive member of the *Streptococcus anginosus* group (1). Despite being part of the normal microbiota, *S. intermedius* is a common pathogen associated with brain abscesses, liver abscesses, and thoracic empyema (2, 3), contributing to the elevated incidence and mortality rates (4). As the available genome sequences are limited on the National Center for Biotechnology Information (NCBI), our knowledge of the genomic factors which contribute to its dissemination to the brain and liver abscess is hindered. Thus, we conducted a detailed genome analysis of *S. intermedius* XH2169.

On 15 August 2021, a 44-year-old woman with septic shock and liver abscesses was admitted to Sir Run Run Shaw Hospital in Zhejiang to undergo intensive care unit (ICU) monitoring. After being admitted to the ICU, percutaneous puncture and drainage of hepatoma were performed in the emergency department on that day. The postoperative drainage was smooth, but the patient suffered from recurrent fever after surgery. *S. intermedius* XH2169 was isolated from the patient’s blood during routine diagnostic analysis (consent for sampling was obtained from the patient) and cultured on a Columbia blood agar plate for 24 hours. Genomic DNA extraction was performed using the QIAamp DNA Mini kit (Qiagen, Germany) and subsequently analyzed on a 1% agarose gel. The identical genomic DNA preparation was employed for both Illumina and Oxford Nanopore Technologies (ONT) sequencing. Illumina sequencing libraries were generated using the Nextflex rapid DNA sequencing kit. For Nanopore sequencing, libraries were conducted by native barcoding expansion set (EXP-NBD104) and SQK-LSK109 ligation sequencing kit without any size selection or trimming. The individual libraries were quantitated using a Qubit version 3.0 fluorometer (Invitrogen, Carlsbad, CA, USA). Finally, the library was loaded onto an R version 9.4.1 flow cell and sequenced on the MinION platform (Oxford Nanopore Technologies, UK).

Whole-genome sequencing was performed on the Illumina HiSeq X Ten platform, as well as on the ONT, UK. The long reads were generated using Guppy v.5.1.2 from ONT (5), with the parameters “–flowcell FLO-MIN106 –kit SQK-LSK109 –barcode_kits ‘EXP-NBD104 EXP-NBD114’,” and the default mode was set to “high accuracy.” Trimmomatic v.0.30 was used to trim the Illumina reads (6). We assembled the filtered MinION reads with Raven v.1.1.10 (7) and subsequently polished the genome sequence with Illumina reads using Polypolish (8). Illumina sequencing achieved an averaged depth of 536×, generating a total of 6,887,896 150-bp paired-end reads. Nanopore sequencing coverage averaged 399×, and a total of 152,875 reads were obtained, with an *N*_50_ value of 8,763. After Polypolish, a total of 1,944,282 bp was obtained (Table 1). A total of 16 rRNAs and 60 tRNAs were identified, as well as three noncoding RNAs. Antibiotic resistance genes were identified using ABRicate v.0.8.13 (https://github.com/tseemann/abricate). The analysis revealed the presence of two resistance genes: *tet*(*M*) and *erm*(*B*). Unless specified, default parameters were utilized for all software. The resulting complete genome sequence was annotated by the NCBI Prokaryotic Genome Annotation Pipeline (http://www.ncbi.nlm.nih.gov/genome/annotation_prok/). For XH2169, the minimum inhibitory concentration (MIC) of penicillin was determined using the broth microdilution method. *Escherichia coli* (American Type Culture Collection) ATCC 25922 was used as quality control. The strain was susceptible to penicillin (MIC = 0.125 µg/mL), according to the Clinical and Laboratory Standards Institute breakpoints (9).

**TABLE 1:** Genome annotation features of *S. intermedius* XH2169^*a*^

Genome features	Value
Genome size (bp)	1,944,282
GC content	37.74
No. of genes (total)	1,966
No. of CDSs (total)	1,891
No. of CDSs (with protein)	1,816
No. of rRNAs	16
No. of tRNAs	60
No. of noncoding RNAs	3
Accession no.	CP136799

^*a*^ GC, guanine-cytosine content; CDS, Coding sequence.

The complete genome sequence of *S. intermedius* XH2169 will promote the understanding of its genetic characterization and offer new insight into liver abscesses caused by this species.

## Data Availability

The whole-genome sequence of XH2169 is available in GenBank under accession number CP136799. The BioSample and BioProject accession numbers are SAMN37717071and PRJNA1025243, respectively. The raw sequence data have been deposited in the Sequence Read Archive under accession numbers SRR26319261 (Nanopore) and SRR26319260 (Illumina).
